# Pure balanced steady‐state free precession imaging (pure bSSFP)

**DOI:** 10.1002/mrm.29086

**Published:** 2021-11-14

**Authors:** Jessica Schäper, Grzegorz Bauman, Carl Ganter, Oliver Bieri

**Affiliations:** ^1^ Department of Biomedical Engineering University of Basel Basel Switzerland; ^2^ Division of Radiological Physics Department of Radiology University Hospital Basel Basel Switzerland; ^3^ Department of Diagnostic Radiology Klinikum rechts der Isar Technical University of Munich Munich Germany

**Keywords:** asymmetry, balanced steady‐state free precession, brain, bSSFP, frequency profile, myelin

## Abstract

**Purpose:**

To show that for tissues the conspicuous asymmetries in the frequency response function of bSSFP can be mitigated by using a short enough TR.

**Theory and Methods:**

Configuration theory indicates that bSSFP becomes apparently “pure” (i.e., exhibiting a symmetric profile) in the limit of TR →0. To this end, the frequency profile of bSSFP was measured as a function of the TR using a manganese‐doped aqueous probe, as well as brain tissue that was shown to exhibit a pronounced asymmetry due to its microstructure. The frequency response function was sampled using N=72 (phantom) and N=36 (in vivo) equally distributed linear RF phase increments in the interval [0,2π). Imaging was performed with 2.0 mm isotropic resolution over a TR range of 1.5–8 ms at 3 and 1.5 T.

**Results:**

As expected, pure substances showed a symmetric TR‐independent frequency profile, whereas brain tissue revealed a pronounced asymmetry. The observed asymmetry for the tissue, however, decreases with decreasing TR and gives strong evidence that the frequency response function of bSSFP becomes symmetric in the limit of TR →0, in agreement with theory. The limit of apparently pure bSSFP imaging can thus be achieved for a TR ∼ 1.5 ms at 1.5 T, whereas at 3 T, tissues still show some residual asymmetry.

**Conclusion:**

In the limit of short enough TR, tissues become apparently pure for bSSFP. This limit can be reached for brain tissue at 1.5 T with TR ∼ 1–2 ms at clinically relevant resolutions.

## 1 INTRODUCTION

The concept of balanced steady‐state free precession (bSSFP) has been first introduced by Carr^1^ in the late 1950s in the context of NMR spectroscopy. Since then, bSSFP has become increasingly popular for imaging due to its high signal efficiency and its $T_{2}/T_{1}$‐weighted contrast.

Although its signal property has been extensively investigated, it was only reported about a decade ago by Miller^2^ that various tissues intrinsically exhibit a strong and rather unexpected asymmetry in the frequency profile of bSSFP. This observed asymmetry in tissues has been associated with local intravoxel frequency distributions driven by its rich and complex underlying microstructure.^3^ Generally, the bSSFP frequency profile, described by Freeman and Hill in an analytical form^4^, is always symmetric around the on‐resonance frequency. However, early work within the context of BOLD MRI, using bSSFP,^5^ pointed out that intravoxel frequency distributions will affect bSSFP’s amplitude–frequency spectrum, but without further elaboration or suggestive remark of a possible resulting asymmetry (although in Figure 3 in Scheffler et al.^5^ an asymmetric bSSFP amplitude–frequency response is shown for a region of interest in the visual cortex). It was argued by Miller^2^, ^3^ that an asymmetric intravoxel frequency distribution will lead to an overall asymmetric bSSFP profile.

Generally, for quantification, any deviation between the measured signal and its presumed underlying analytical form, such as an asymmetric frequency response function, will lead to a bias in the corresponding parameter estimates. The question, whether the regime of symmetric frequency distributions can be reached, is thus of fundamental interest, especially within the context of quantitative MRI.

In this work, we use configuration theory^6^ to show that, generally, tissue “forgets” about its spectral composition when using bSSFP imaging in the limit of TR →0. As a result, the frequency response function of bSSFP should become apparently “pure” (in the sense of imitating a homogeneous, symmetric frequency distribution) for short enough TR. It is shown that this limit of apparent symmetry can be reached for brain tissue for repetition times below about 1‐2 ms at 1.5 T. Therefore, pure bSSFP imaging of the brain is generally possible while retaining a clinically relevant resolution.

## 2 THEORY

We consider a voxel, consisting of multiple tissue components. These components (labeled by *k*) usually differ in their tissue properties ($T_{1,k},T_{2,k},\ldots$) and frequency distributions $p_{k}\left(\omega \right)$. The average frequency for each component ${\left\langle \omega \right\rangle }_{k}:=\int d\omega p_{k}\left(\omega \right)\omega$ depends on the chemical shift $\delta_{k}$ and the off‐resonance over the voxel. The spectral shape of $p_{k}\left(\omega \right)$ defines the line width via homogeneous ($T_{2,k}$) and inhomogeneous ($T_{2,k}^{\prime }$) broadening of the linewidth. Finally, the relative weights $\zeta_{k}$ of the peaks are defined as $\zeta_{k}=\int d\omega p_{k}\left(\omega \right)$ and may be considered as normalized, $\sum_{k}\zeta_{k}=1$.

Using configuration theory ^6^, we may then write the bSSFP signal for any given RF phase increment ϕ (used to sample the profile) immediately after excitation as

(1) $$\begin{array}{c} M\left(\phi ,\mathrm{TR}\right)=\sum_{k}\int d\omega p_{k}\left(\omega \right)\sum_{n}e^{in(\phi ‐\omega \mathrm{TR})}m_{k}^{\left(n\right)} \end{array}$$

where *n* is the coherence pathway order and the $m_{k}^{\left(n\right)}$ depend on TR, $T_{1,k}$, $T_{2,k}$ and the flip angle α.

We immediately see that (note that the actual TE < TR does not matter in the limit TR→0)

(2) $$\begin{array}{c} \lim_{\mathrm{TR}\to 0}M\left(\phi ,\mathrm{TR}\right)=\sum_{k}\zeta_{k}\sum_{n}e^{in\phi }m_{k}^{\left(n\right)}=\sum_{n}e^{in\phi }M^{\left(n\right)}=:M\left(\phi \right) \end{array}$$

This means the voxel eventually “forgets” about its spectral composition, having the apparent properties of a “pure” voxel. Intuitively, this relates to the fact that for any pair of tissue components a reduction of TR translates to a reduced average phase difference accumulation, $\Delta \theta_{\mathrm{ij}}:=\left({\left\langle \omega \right\rangle }_{i}‐{\left\langle \omega \right\rangle }_{j}\right)\cdot \mathrm{TR}=2\pi \gamma B_{0}\delta_{\mathrm{ij}}\mathrm{TR}$, where $\delta_{\mathrm{ij}}=\delta_{i}‐\delta_{j}$. For the 2π periodicity of the bSSFP frequency response function the “purity” condition

(3) $$\begin{array}{c} 2\pi \gamma B_{0}\delta_{\mathrm{ij}}\mathrm{TR}\approx 0 \end{array}$$

implies

(4) $$\begin{array}{c} \delta_{\mathrm{ij}}\ll \frac{1}{\gamma B_{0}\mathrm{TR}}\;\text{or}\;\mathrm{TR}\ll \frac{1}{\gamma B_{0}\delta_{\mathrm{ij}}} \end{array}$$

Notably this becomes less stringent with decreasing $B_{0}$ for fixed $\delta_{\mathrm{ij}}$.

Generally, the complexity of the underlying tissue microstructure determines the minimum number of $\delta_{\mathrm{ij}}$ required for an appropriate modeling of the tissue. For myelin, it was shown that three microcompartments are enough to describe the frequency profile adequately.^7^ Moreover, $\delta_{\mathrm{ij}}$ was shown to be orientation dependent,^7^, ^8^, ^9^ with a perpendicular orientation to $B_{0}$ exhibiting larger frequency offsets. Values of $\delta_{\mathrm{ij}}∼0.1\text{‐}0.2$ ppm are typically reported.^3^, ^8^, ^9^ For 20 Hz at 3 T,^3^ this leads to a theoretical limit of TR ≪ 50 ms. Consequently, for 1.5 T one obtains a theoretical limit of TR ≪ 100 ms. Generally, one can assume that, in practice, the used TR has to be 1‐2 orders of magnitude smaller than this calculated upper bound in order to fulfill the condition.

Part of the present work is to examine which TR sufficiently fulfills this limit. On modern clinical scanners a TR of 1‐2 ms can generally be reached for reasonable resolutions. It is expected that this suffices to test the theory.

## 3 METHODS

Imaging was performed at 3 T (MAGNETOM Prisma, Siemens Healthineers, Erlangen, Germany) and 1.5 T (MAGNETOM AvantoFit, Siemens Healthineers, Erlangen, Germany) using a 3D Cartesian ultra‐fast bSSFP sequence.^10^ All data analysis and visualization were done using Matlab R2019a (The MathWorks, Inc., Natick, MA). Scanning was approved by the local ethics committee and written informed consent was given by all volunteers beforehand.

Scans were done for monodisperse spin densities using a manganese‐doped, aqueous phantom ($T_{1}=860$ ms, $T_{2}=70$ ms at 3 T and $T_{1}=860$ ms, $T_{2}=100$ ms at 1.5 T) and for in vivo brain where a strong profile asymmetry is expected.^3^ A nominal flip angle of $\alpha =10^{\circ }$ was chosen for four different values of TR (1.5, 3.0, 5.0, and 8.0 ms) with corresponding bandwidths of {1775, 610, 270, 150} Hz/Px at 3 T and {1860, 620, 270, 150} Hz/Px at 1.5 T. The echo was symmetric for each TR, yielding TE = TR/2. A slab‐selective sinc pulse with a time‐bandwidth‐product of 2.0 and a duration of 210 μs (1.5 ms), 360 μs (3 ms), 340 μs (5 ms), 420 μs (8 ms) at 3 T and 200 μs (1.5 ms), 320 μs (3 ms), 280 μs (5 ms), 380 μs (8 ms) at 1.5 T was used. The different pulse lengths were chosen to ensure the exact desired values of TR for better comparability between the two field strengths. A field‐of‐view of 256 mm with a base resolution of 128, 75% resolution in phase direction and a slice thickness of 2.0 mm were chosen, resulting in an imaging matrix of 128×96×80 and an isotropic resolution of 2.0 mm. For each TR setting, *N* scans with equally distributed linear RF phase increments in the interval [0,2π) were recorded. In order to ensure steady‐state conditions, dummy preparation periods of 7.5 s (TR = 1.5 ms and TR = 3 ms), 5 s (TR = 5 ms) and 4 s (TR = 8 ms) for the phantom scans and 15 s (TR = 1.5 ms and TR = 3 ms), 12.5 s (TR = 5 ms) and 4 s (TR = 8 ms) for the in vivo scans were used for each phase‐cycle. The phantom scans were performed with N=72, the brain scans with N=36. Scanning took 19:50 min (TR = 1.5 ms), 30:40 min (TR = 3 ms) 38:06 min (TR = 5 ms) and 53:45 min (TR = 8 ms) for the phantom and 14:25 min (TR = 1.5 ms), 19:50 min (TR = 3 ms), 22:48 min (TR = 5 ms) and 26:53 min (TR = 8 ms) for the brain scans to complete. A summary of all relevant scan parameters can be found in Supporting Information Table S1 and S2. For better comparison, scans were performed on the same volunteer at 3 and 1.5 T. The data was coregistered afterwards in Matlab. Measurements for all values of TR were done in the same run at each field strength.

For the brain and phantom scans, the asymmetry index (AI) was calculated as

(5) $$\begin{array}{c} \text{AI}=\frac{h_{p}‐h_{n}}{\max\left(h_{p},h_{n}\right)‐h_{c}} \end{array}$$

where $h_{p,n}$ is the peak signal at the positive (*p*) or negative (*n*) frequency offset from the off‐resonance and $h_{c}$ is the minimal stop‐band value. This is a slightly modified version of the formula used by Miller et al.^3^ Scaling the peak height difference with the total variation of the profile accounts for TR‐related variations, and thus offers a better comparability between different TRs. For the calculation, the stop‐band position was located and centered. Then a maximum search was carried out on both sides of the stop‐band to locate $h_{p}$ and $h_{n}$.

## 4 RESULTS

During the experiments, a slight thermal drift of the frequency was noticed. Consequently, the profiles do not perfectly match the (0,2π) interval. Since the drift is slow and weak, especially for small TR, it is not expected that this has a significant influence on the symmetry of the profile. Therefore, it is not accounted for.

Moreover, the shown profiles were not centered and the stop‐band shift represents the local off‐resonance, which is unrelated to the AI.

**FIGURE 1:**
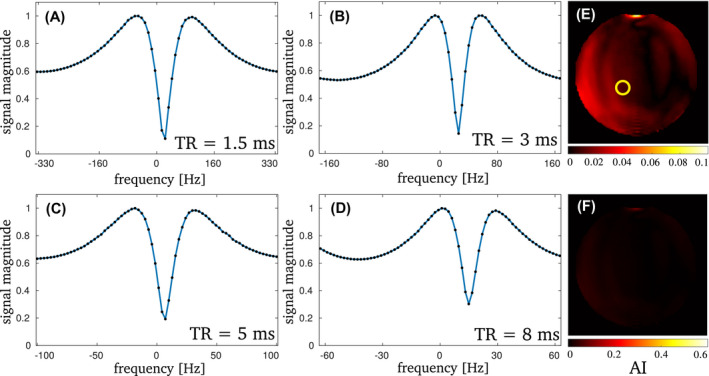
The bSSFP profiles for the phantom scans with TR ={1.5,3,5,8} ms at 3 T are shown in (A)–(D). The profiles were normalized to their maximum peak height for better visibility of the relative scale of the peak and stopband values. The AI is less than 0.02 for all shown profiles. On the right AI maps of the phantom midsection are shown for TR = 8 ms. In (E) the map is scaled to visible values. The circle indicates the region from which the averaged profiles were taken. The averaging was done over 3×3 pixels. For better comparison, (F) shows the identical map, scaled to the range which is used for the in vivo data in Figure 3. No considerable asymmetry can be observed, with a maximum value of AI = 0.04 at the edge of the phantom due to surface effects. The phase‐cycled data sets each had an SNR of 175 (TR = 1.5 ms), 330 (TR = 3 ms) and 350 (TR = 5 and TR = 8 ms), respectively

For the phantom scans, the observed frequency response of bSSFP is symmetric for all TR values investigated at 3 and 1.5 T, as can be expected for pure voxels with symmetric frequency dispersions. Example profiles are shown for 3 T in Figure 1. The observed asymmetry is less than 0.02 for all shown profiles, as well as for the whole midsection of the phantom. Toward the edge of the phantom the asymmetry increases slightly, due to surface effects and pixel size. The mean AI over the whole phantom is 0.025 ± 0.013 for all TR.

**FIGURE 2:**
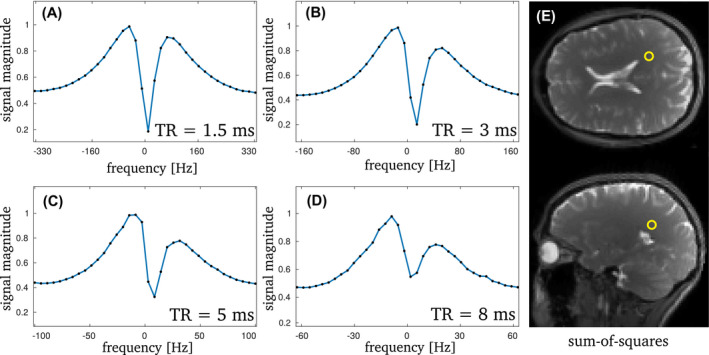
The 3 T bSSFP profiles for the mean value of a WM ROI are displayed in (A)–(D). The according ROI (3×3 pixels) is shown in (E) for two orientations. A clear decrease in asymmetry is visible in the profiles for decreasing TR. The corresponding values for the AI are: 0.09 (TR = 1.5 ms), 0.21 (TR = 3 ms), 0.32 (TR = 5 ms) and 0.39 (TR = 8 ms). This general trend is observed over the whole brain, but is strongest in WM. The phase‐cycled data sets each had an SNR of 40 (TR = 1.5 ms and TR = 3 ms), 100 (TR = 5 ms) and 115 (TR = 8 ms), respectively

**FIGURE 3:**
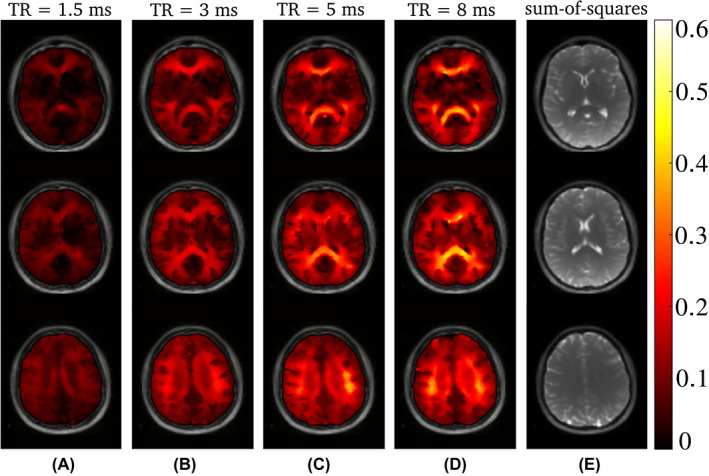
The calculated AI maps for the different TR are shown in (A)–(D) for three axial slices of the in vivo brain scans at 3 T. It is clearly visible that, overall, the values for TR = 1.5 ms are smaller than for higher TR. However, some asymmetry is still present. (E) shows the anatomical reference for each slice

This is in contrast to the findings in tissues, such as for brain white matter (WM). Figure 2 shows bSSFP frequency response profiles for a selected region of interest in WM at 3 T. A pronounced asymmetry (AI = 0.39 at TR = 8 ms and AI = 0.32 at TR = 5 ms) is observed for high TR values; in agreement with previous findings.^3^ The asymmetry index, however, markedly decreases with a reduction in TR: at TR = 1.5 ms the asymmetry is as low as 0.09, but still noticeable. Example AI maps are shown in Figure 3 for three axial slices, along with a morphological image as reference. As reported by Miller et al.^3^ a rich variation of the AI is observed for brain tissue, with highest AI values in the corpus callosum (AI ∼ 0.56 at 8 ms), while the region around the corticospinal tract exhibits generally the lowest asymmetry (AI ∼ 0.1 at 8 ms). Overall, the AI maps show a pronounced sensitivity on the TR and decrease with decreasing TR, as expected from theory.

**FIGURE 4:**
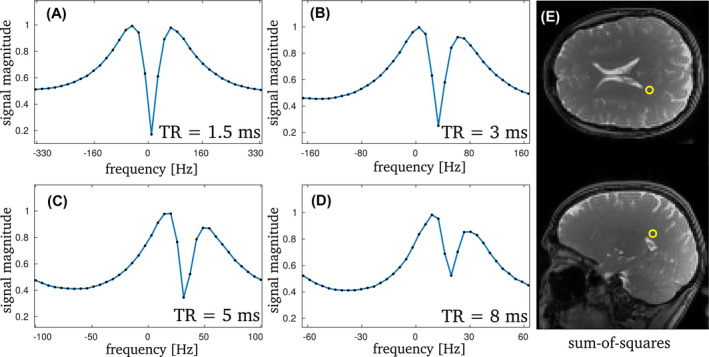
The 1.5 T bSSFP profiles for the mean value of a WM ROI are displayed in (A)–(D) with the according 3×3 pixel ROI shown in (E). Again, the clear decrease in asymmetry is visible in the profiles for decreasing TR. Overall, the asymmetry is lower than at 3 T. The corresponding values for the AI are: 0.01 (TR = 1.5 ms), 0.08 (TR = 3 ms), 0.15 (TR = 5 ms) and 0.18 (TR = 8 ms). The phase‐cycled data sets each had an SNR of 20 (TR = 1.5 ms), 30 (TR = 3 ms), 40 (TR = 5 ms) and 50 (TR = 8 ms), respectively

**FIGURE 5:**
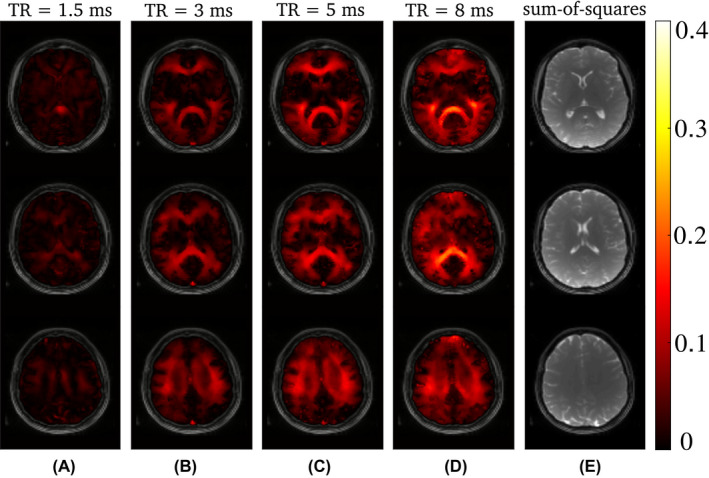
The calculated AI maps for the different TR are shown in (A)–(D) for three different axial slices of the in vivo brain scans at 1.5 T. Again, a clear decrease in the AI toward lower TR is visible. At TR = 1.5 ms, only small residue asymmetry is visible. (E) show the anatomical reference. Note that the colour scale is different than for 3 T

From Equation (4), the purity condition suggests that the minimum required TR to achieve an apparent symmetric bSSFP frequency response also directly relates to the main magnetic field strength. In analogy to the observations at 3 T, a pronounced asymmetry is observed at 1.5 T for long TR which decreases rapidly with decreasing TR (Figure 4). In contrast, however, the purity condition is reached for TR ∼ 1–2 ms (Figure 5). This can be seen as a soft threshold of pure bSSFP, since absolute asymmetry is not achievable. Moreover, for equal TR a generally lower asymmetry is observed at 1.5 T as compared to 3 T.

## 5 DISCUSSION

The above results clearly support the presented theory, showing that for brain tissue the observed asymmetry of the bSSFP frequency profile (i) is modulated by $B_{0}$ and TR, (ii) decreases with decreasing TR and (iii) vanishes in the limit of TR →0.

Several studies have reported bSSFP signal deviations from the Freeman–Hill formulae, for example, due to water‐exchange,^11^ diffusion,^12^ magnetization transfer,^13^ finite RF pulse effects,^14^ or asymmetric frequency response functions.^2^ Generally, a symmetric frequency response is presumed within the context of relaxometry using multiple phase‐cycled bSSFP scans.^15^, ^16^, ^17^ It is evident that any deviation between the presumed underlying theory (relying on a symmetric frequency distribution) and the observed asymmetric signal behaviour (emanating from an asymmetric frequency distribution) will lead to a bias in the corresponding model parameter estimates. Several studies pointed out that especially $T_{1}$ of WM brain tissue deviates substantially from expectations when using bSSFP.^15^, ^16^, ^17^ This effect was attributed to tissue microstructure leading to asymmetric frequency distributions which in turn lead to an asymmetric frequency response function.^3^ A possible approach to remove this bias for a specific measurement setup was recently suggested using neural networks.^18^ From a methods point‐of‐view, however, scanning is preferentially being performed in a regime where theory and measurements match and voxels forget about their intrinsic spectral composition rather than learning a specific bias away. The present study shows that this can be achieved when chosing the right setting.

Generally, the symmetry limit is reached earlier at 1.5 T as compared to 3 T, as expected from the $B_{0}$ dependence of Equation (4). For the shortest TR explored, some asymmetry was still present at 3 T for TR = 1.5 ms, while at 1.5 T only small residue asymmetry was observed. It is expected that the symmetry limit can be reached only for TR < 1 ms at 3 T, which can only be obtained to the detriment of resolution.

This indicates that especially on low‐field scanners symmetric profiles can, in general, be expected for rather common TR and reasonable resolution. Due to the current trend towards low‐field MRI,^19^ pure bSSFP imaging should, therefore, be feasible on such systems. Conversely, on ultra‐high field scanners chemical‐shift related frequency offsets (discussed below) will be even stronger, making pure bSSFP imaging impossible on these systems. See, eg., a study by Ehses et al.^20^ where a rather strong asymmetry was observed for TR = 4 ms at 9.4 T.

As can be seen from Equation (4), the dependence on TR and the according limit of TR →0 are influenced by the chemical shift $\delta_{k}$ of the underlying frequency distribution from the on‐resonance frequency. For brain tissue, where myelination is the probable cause of dispersion, the empirical frequency shift of 20 Hz at 3 T^3^ was used, corresponding to a chemical shift of 0.15 ppm. This leads to the relatively generous limit of TR ≪ 50 ms for 3 T (TR ≪ 100 ms at 1.5 T). Aside from brain tissue, a prominent asymmetry is observed in muscle tissue where partial volume effects due to fat deposits and blood vessels could be the underlying cause.^2^ At 3 T the water–fat chemical shift of 3.5 ppm amounts to 440 Hz (220 Hz at 1.5 T). This leads to the asymmetry condition TR ≪ 2 ms at 3 T (TR ≪ 4 ms at 1.5 T), which is not feasible. In this case, it is not possible to reach a symmetric profile. Furthermore, the effect in water‐fat voxels is expected to be dependent on voxel size due to the fact that larger voxels are more likely to contain both, water and fat.^2^ This is in contrast to brain tissue where microscopic effects cause the asymmetry, making it mostly independent of voxel size.^3^

While pure bSSFP could offer an opportunity to improve reliable quantitative MRI, the asymmetry of the profile also offers a source of microstructural contrast. The AI depends on the chemical exchange^21^ and the tract orientation relative to the $B_{0}$ field.^3^, ^7^, ^9^, ^20^ Hence, the high AI values in the corpus callosum (perpendicular to $B_{0}$) and low values in the corticospinal tract (parallel to $B_{0}$). In this context, it might be noteworthy, that in our work, selective RF pulses were used. Thus, the flip angle varies over the slab. While this can influence the absolute value of the asymmetry, it does not affect the overall trend of decreasing asymmetry, which was the main focus of this work.

It is clear that these two applications, pure quantitative imaging and AI as a contrast, oppose each other. While quantitative MRI could potentially benefit from pure bSSFP at low fields and small TR, the AI contrast becomes strongest at high fields and, within the frame of bSSFP, long TR. This has to be taken into account when planning for experiments.

Regardless of the application, this study shows that the AI is very dependent on external parameters, namely $B_{0}$ and the TR. This stands in contrast to other intrinsic tissue properties, such as diffusion, which is independent of most external factors and can be used as a reliable diagnostic measure. Due to this dependence, it is crucial to perform measurements with equal parameters in order to get comparable results.

## 6 CONCLUSION

We have shown that the pronounced asymmetry in the bSSFP frequency response profile vanishes for TR →0 and is, in general, $B_{0}$ dependent. This supports the assumption that the asymmetric profile has its origin in intravoxel frequency distributions. At 3 T, the asymmetry does not completely vanish for the investigated minimum value of TR = 1.5 ms, whereas at 1.5 T, symmetric profiles are already reached at TR ∼ 1.5 ms. Therefore pure bSSFP imaging becomes feasible for clinically relevant resolutions when using low field strengths and short TR.

## Supporting information


**TABLE S1** General parameters of the used Cartesian ultra‐fast 3D bSSFP sequence
**TABLE S2** Sequence parameters for the different scans, divided accordingly for the phantom and in vivo scans and the two field strengths
