# Supplementary material for: Pure balanced steady‐state free precession imaging (pure bSSFP)
Source: Magn Reson Med. 2021 Nov 14;87(4):1886–93. doi: 10.1002/mrm.29086 (PMC9299476; doi:10.1002/mrm.29086)
Supplement: Supplementary file 1 — TABLE S1 General parameters of the used Cartesian ultra‐fast 3D bSSFP sequence TABLE S2 Sequence parameters for the different scans, divided accordingly for the phantom and in vivo scans and the two field strengths [file MRM-87-1886-s001.pdf]

Pure balanced steady-state free precession imaging (pure bSSFP)

Supporting Material

Jessica Schäper[1,2], Grzegorz Bauman[1,2], Carl Ganter[3], and Oliver Bieri[1,2]

[1]Division of Radiological Physics, Department of Radiology, University Hospital Basel, University of Basel, Basel, Switzerland.

[2]Department of Biomedical Engineering, University of Basel, Basel, Switzerland.

[3]Department of Diagnostic Radiology, Klinikum rechts der Isar, Technical University of Munich, Munich, Germany

α	10°
FOV	256×192×160 mm^3
matrix	128×96×80
resolution	2.0 mm iso
tbw product	2.0
phase-cycles	72 (phantom)<br>36 (in vivo)

Supporting Information Table S1: General parameters of the used Cartesian ultra-fast 3D bSSFP sequence.

	3 T, phantom				3 T, in vivo				1.5 T, in vivo			
TR [ms]	1.5	3.0	5.0	8.0	1.5	3.0	5.0	8.0	1.5	3.0	5.0	8.0
bandwidth [Hz/px]	1775	610	270	150	1775	610	270	150	1860	620	270	150
pulse length [μs]	210	360	340	420	210	360	340	420	200	320	280	380
dummy length [s]	7.5	7.5	5	4	15	15	12.5	4	15	15	12.5	4
scan time [min]	19:50	30:40	38:06	53:45	14:25	19:50	22:48	26:53	14:25	19:50	22:48	26:53

Supporting Information Table S 2: Sequence parameters for the different scans, divided accordingly for the phantom and in vivo scans and the two field strengths. For all scans TE = TR/2.
